# A Screening Method for Assessing Cumulative Impacts

**DOI:** 10.3390/ijerph9020648

**Published:** 2012-02-16

**Authors:** George V. Alexeeff, John B. Faust, Laura Meehan August, Carmen Milanes, Karen Randles, Lauren Zeise, Joan Denton

**Affiliations:** Office of Environmental Health Hazard Assessment, California Environmental Protection Agency, 1515 Clay Street, 16th Floor, Oakland, CA 94612, USA; Email: john.faust@oehha.ca.gov (J.B.F.); Laura.August@oehha.ca.gov (L.M.A.); carmen.milanes@oehha.ca.gov (C.M.); karen.randles@oehha.ca.gov (K.R.); lauren.zeise@oehha.ca.gov (L.Z.); joandenton@comcast.net (J.D.)

**Keywords:** cumulative impacts, cumulative risk assessment, environmental justice, community health

## Abstract

The California Environmental Protection Agency (Cal/EPA) Environmental Justice Action Plan calls for guidelines for evaluating “cumulative impacts.” As a first step toward such guidelines, a screening methodology for assessing cumulative impacts in communities was developed. The method, presented here, is based on the working definition of cumulative impacts adopted by Cal/EPA [1]: “*Cumulative impacts means exposures, public health or environmental effects from the combined emissions and discharges in a geographic area, including environmental pollution from all sources, whether single or multi-media, routinely, accidentally, or otherwise released. Impacts will take into account sensitive populations and socio-economic factors, where applicable and to the extent data are available.*” The screening methodology is built on this definition as well as current scientific understanding of environmental pollution and its adverse impacts on health, including the influence of both intrinsic, biological factors and non-intrinsic socioeconomic factors in mediating the effects of pollutant exposures. It addresses disparities in the distribution of pollution and health outcomes. The methodology provides a science-based tool to screen places for relative cumulative impacts, incorporating both the pollution burden on a community- including exposures to pollutants, their public health and environmental effects- and community characteristics, specifically sensitivity and socioeconomic factors. The screening methodology provides relative rankings to distinguish more highly impacted communities from less impacted ones. It may also help identify which factors are the greatest contributors to a community’s cumulative impact. It is not designed to provide quantitative estimates of community-level health impacts. A pilot screening analysis is presented here to illustrate the application of this methodology. Once guidelines are adopted, the methodology can serve as a screening tool to help Cal/EPA programs prioritize their activities and target those communities with the greatest cumulative impacts.

## 1. Introduction

Many Californians live in close proximity to multiple sources of pollution. Past industrial, agricultural and mining activities have left a toxic legacy near many communities of the state. In addition, rail yards, freeways, ports, and other facilities bring together vehicles and equipment that produce emissions from diesel fuel and gasoline. Today, communities by these locations are predominantly low-income, often with a large percentage of racial and ethnic minorities and non-English speakers [2,3]. Like other low-income communities, they face additional challenges that can affect their health, including limited access to health care, poor nutrition, shortage of grocery stores, and a lack of parks and open space.

Recognizing the need to address these inequities, California enacted a law mandating that its environmental programs address environmental justice (EJ). The law defines EJ as “the fair treatment of people of all races, cultures, and incomes with respect to the development, adoption, implementation and enforcement of environmental laws, regulations and policies” [4]. California Environmental Protection Agency’s (Cal/EPA) Environmental Justice Action Plan [1] identified the development of methods and policies involving cumulative impacts analyses as a key step toward addressing EJ. Cal/EPA adopted a working definition of cumulative impacts as:

“The exposures, public health or environmental effects from the combined emissions and discharges in a geographic area, including environmental pollution from all sources, whether single or multi-media, routinely, accidentally, or otherwise released. Impacts will take into account sensitive populations and socio-economic factors, where applicable and to the extent data are available.” [1].

The scientific foundation for addressing cumulative impacts is based on evidence of: (1) the relationship between environmental pollution and health effects; (2) disparities in exposures and environmental conditions; (3) differences in intrinsic and socioeconomic (non-intrinsic) sensitivity to pollutants; and (4) health disparities among various segments of the population. 

Converging lines of evidence reinforce concern for the cumulative impact of pollutants, particularly in low-income and minority communities. Communities in highly polluted locations are often predominantly low-income, with a large percentage of racial and ethnic minorities and non-English speakers. Certain populations may be especially sensitive to environmentally mediated disease due to *intrinsic* characteristics such as age, pre-existing health conditions, gender and genetics. *Non-intrinsic* factors such as income level and race/ethnicity may also modify the response to pollutant-mediated adverse effects. 

Differences exist between segments of the population in health outcomes known to be influenced or caused by environmental pollutants [5]. For example, in one study, low-income African-American mothers exposed to traffic-related air pollution had twice the chances of delivering a preterm baby as white women [6]. Another study found reduced birth weight from exposure to particulate pollution (PM 2.5) was greater among offspring of black mothers compared to white mothers [7]. Studies in California and elsewhere show that low-income people have higher rates of asthma symptoms and hospitalizations [8,9,10]. 

Disparities in cancer and cardiovascular disease are well-established across various socioeconomic and racial and ethnic groups. Among major racial groups in the U.S., cancer incidence is highest among African Americans for lung, colon, prostate and all cancer sites combined [11]. Higher socioeconomic status, as measured by educational attainment, income, and poverty level, is associated with lower prevalence of cardiovascular disease and risk factors such as smoking and diabetes [12,13].

Environmental programs aim to protect public health and the environment from the adverse effects of toxic and hazardous contaminants and other harmful agents. Current environmental regulations are generally established to set limits for individual pollutants in air, water, soil, food or other sources of exposure. While few originally accounted for exposure to multiple pollutants from multiple sources, over the last 20 years, environmental policies have been evolving to incorporate impacts of multiple sources or multiple pollutants. Risk assessments conducted for cleanups of contaminated sites were among the first to include multiple chemicals. However there are concerns overall over the extent of the multi-chemical analysis, and the consideration given, if any, to non-chemical stressors such as those that may influence vulnerability (for example, poverty or race/ethnicity). The U.S. Environmental Protection Agency’s (U.S. EPA) 2003 *Framework for Cumulative Risk Assessment* demonstrates the need for cumulative risk assessments to take into account multiple agents and stressors [14], including non-chemical ones. A recent National Research Council report calls for a fresh approach to adequately address cumulative impacts and highlights a need for simplified risk assessment tools that weigh nonchemical stressors, the population vulnerability to pollution, and background risk factors [15]. Here, we have used the term “impacts” to broadly represent the contributions to impact described in the Cal/EPA working definition and an assessment approach that brings together a diverse set of information, both quantitative and semi-quantitative. The term “cumulative risk” is taken to be a more quantifiable approach to assessment. 

Approaches to assessing and mitigating cumulative impacts are a logical next step in applying the best available science to environmental protection. The development of cumulative impacts analysis tools can improve and enhance the overall ability to identify environmental concerns in a community. Such analyses can support protective actions and ensure that resources are directed where they will provide the greatest benefit.

In 2010, Cal/EPA released a report that presents a framework for developing a screening methodology to evaluate the cumulative impacts of multiple sources of pollution in specific communities or geographic areas [16]. Applying the proposed Cal/EPA screening methodology [16] is an initial step for integrating cumulative impacts considerations into decision-making, filling a gap that inhibits the achievement of environmental justice. The feasibility of this methodology is demonstrated by a pilot screen that analyzes relative cumulative impacts across selected California communities. The proposed Cal/EPA screening methodology and pilot analysis are presented in this paper. 

## 2. Methodology

### 2.1. Overview

**Figure 1:**
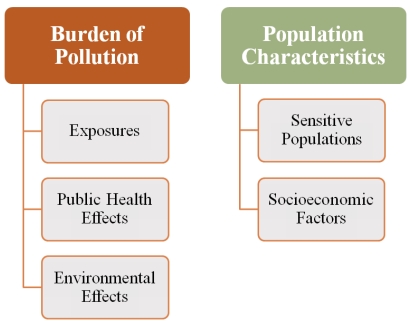
The five components of cumulative impact.

The screening methodology was developed based on Cal/EPA’s working definition of cumulative impacts, and in consideration of existing approaches for assessing impacts on communities. This methodology screens for relative levels of cumulative impacts among communities using a scoring system based on five primary components identified from the working definition: exposures, public health and environmental effects, sensitive populations and socioeconomic factors (see Figure 1). Scores are calculated for a population of interest in a given geographic area—that is, spatial boundaries delineated by a residential area, a school site or other geopolitical subdivision, or defined by the margins of the area where the population works or lives (as in the case of migrant farm workers).

As shown in Figure 1, the five components are divided into two main groups: *pollution burden* (pollution-related components in a geographic area) and *population characteristics* (factors related to intrinsic and non-intrinsic characteristics of the people living in the geographic area). The benefit of separating the components into these groups becomes evident when calculating cumulative impacts, as discussed below in the analytical approach.

### 2.2. Analytical Approach

**Figure 2:**
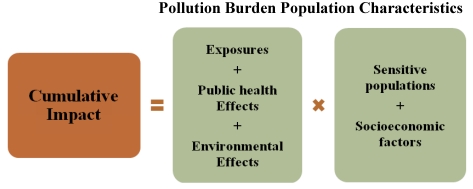
Formula for estimating relative cumulative impact.

An overall cumulative impact score for an area is calculated by multiplying its pollution burden score—the sum of the scores for exposures, public health effects and environmental effects—by its population characteristics score—the sum of the scores for sensitive population and socioeconomic factors (see Figure 2). This multiplicative approach is consistent with existing risk assessment guidance addressing sensitive populations. For example, risk assessments carried out under the Food Quality Protection Act of 1996 use a multiplier to account for special sensitivities of children. When accounting for age-specific sensitivity to carcinogens in cancer potency calculations, recent assessments by the U.S. Environmental Protection Agency and by California’s Office of Environmental Health Hazard Assessment also apply a multiplier [17,18]. Evidence from human studies have shown that population characteristics can modify multiplicatively the response to pollution burden, providing scientific support for the use of a multiplier [6,19].

A range of possible scores is assigned to each component, as shown in Table 1. The overall cumulative impact score ranges from 6–120, a range large enough to distinguish impacts among communities. 

**Table 1:** Range of scores assigned for each component.

	Component	Range of Possible Scores
**Pollution burden**	Exposures	1–10
	Public health effects	1–5
	Environmental effects	1–5
**Population characteristics**	Sensitive populations	1–3
	Socioeconomic factors	1–3
	**Cumulative impact**	**6–120**

The range of scores for each component was set based on several factors. Among the pollution burden components, the maximum possible value assigned reflected the strength of the available data—including considerations of data quality and quantity—and Cal/EPA’s ability to address its impact. Since there is considerable information available on the types and extent of potential exposures within a community due to extensive monitoring and reporting systems established in California, this component was assigned a maximum value of 10. Further, exposures are most closely associated with pollution impact and are the most likely to be addressed by environmental programs. On the other hand, there is less certainty and less information on the other two pollution burden components. There is less certainty about how much of a specific public health effect or an environmental effect was caused by pollution. For example, asthma, while known to be linked to environmental pollution, is also influenced by intrinsic factors such as genetics or non-intrinsic factors such as access to medical care. Further, less standardized information is available on these two components at the community level. Hence, they were assigned a maximum value of 5. 

Each of the population characteristic components is assigned a maximum value of 3. This is based on scientific evidence for a several-fold difference in response to environmental pollutants among certain populations based on either intrinsic (biological and physiological traits) or extrinsic (socioeconomic status and race/ethnicity) factors [6,19]. 

### 2.3. Indicators

One or more indicators are selected to represent each component in order to arrive at a score. Indicators are simple measures that provide information about the condition of the community with respect to the component it represents. Example indicators are as follows: for exposures, ambient environmental concentrations, chemical emissions, chemical releases, and chemical use inventories; for public health effects, incidence and prevalence of disease caused or exacerbated by exposure to pollutants; for environmental effects, extent of ecological damage, ecological changes, presence of waste or contamination, and threat of accidental chemical releases; for sensitive populations, life-stage, age, and disease state; and for socioeconomic factors, income and educational attainment.

Each indicator is assigned a score within the range for its component. An indicator score for each community reflects that community’s ranking across the state. For example, communities are scored from highest to lowest based on the level of ozone in the air (representing exposure). The scores are then divided into 10 equal subgroups: communities having the lowest ozone levels receive a score of 1, and those with the highest ozone levels, a score of 10. Thus, one-tenth of the communities are assigned each score. When multiple indicators represent a component, the average of the indicator values becomes the score for that component. 

Criteria will be developed to guide indicator selection. The goal is to select as few indicators for each component as possible to adequately describe the relative magnitude of that component’s impact in a community. The possibility that two or more indicators may represent the same or similar contribution to impact needs to be examined (through statistical correlations, conceptual models exploring causal relationships or other means). Some level of overlap may be unavoidable due to limitations to available data or scientific uncertainty. To allow the ranking of communities statewide, and to be transparent, publicly available statewide data must be available for each indicator. 

### 2.4. Pilot Analysis

To demonstrate the application of the screening methodology, a pilot analysis was conducted to calculate cumulative impact scores for 30 diverse communities from different parts of California. Indicators were selected to represent each component (see Table 2). For purposes of the pilot analysis, the selected indicators are intended to be illustrative of the process only, and may not necessarily reflect the best choices. 

Data for the indicators were obtained from existing publicly available statewide databases. In this analysis, the geographic areas selected to approximate communities of interest were delineated using ZIP codes boundaries. The use of ZIP codes is intended to help make the analysis more meaningful to the general public. The ZIP code was also selected for practical reasons, including the availability of data in this geographical unit. For example, most of the health data used in the pilot are available only by ZIP code. The 30 ZIP codes in this analysis represent demographically diverse communities in various geographic regions of California. In presenting the results here, the specific ZIP codes have been masked to avoid mischaracterizing community impacts, given that the methodology is still being refined and the analysis is only meant as a demonstration. 

**Table 2:** Indicators chosen for the pilot cumulative impact analysis.

Component	Contribution to Component	Indicator	Data Source
**Exposures**	Emission of fine particles	PM 2.5 concentrations	California Air Resources Board: California Air Quality Data
	Criteria Air Pollutants	Ozone concentrations	
	Emissions and discharges of hazardous chemicals	Toxic releases from industrial facilities	U.S. EPA
	On road mobile sources	Traffic volumes	California Environmental Health Tracking Program
	Pesticides	Pesticide use	California Department of Pesticide Regulation
**Public Health Effects**	Birth outcomes	Low birth weight rate	California Department of Public Health
	Disease rates with environmental component	Heart disease mortality rate	
	Cancer rates with environmental component	Cancer mortality rate	
	Asthma	Asthma hospitalization rate	California Environmental Health Tracking Program
**Environmental Effects**	Hazardous waste sites & Brownfields	Hazardous waste & clean-up sites	California Department of Toxic Substances Control
	Spills, leaks	Leaking underground fuel tanks	California State Water Resources Control Board
**Sensitive Populations**	Presence of children	Percent under age 5	U.S. Census
	Presence of elderly	Percent over age 65	
**Socioeconomic Factors**	Educational attainment	Percent with less than high school education	U.S. Census
	Income level	Median household income	
	Poverty	Percent residents below 2x national poverty level	

We determined the range and distribution of values for each indicator, based upon available data, with the goal of using complete California distributions. The ZIP codes were scored based upon where its indicator value occurred in the available distribution of values. The score corresponds to a value within the range of possible scores for the component which the indicator represents (as described in Table 1). Thus, each indicator for each ZIP code was assigned a score of 1 to 10 for exposure, 1 to 5 for public health effects and environmental effects, and 1 to 3 for sensitive populations and socioeconomic factors. For example, ZIP codes will be subdivided into ten scores for an indicator of the exposure component. A score of 10 would be assigned to a ZIP code that ranked within the highest 10th (*i.e.*, in the 90th percentile) of all communities; a score of 1 would be assigned to a ZIP code that ranked within the lowest 10th (*i.e.*, in the 10th percentile). The scores for all indicators representing a component were averaged to calculate the component’s score. 

Finally, a cumulative score was calculated for each ZIP code following the formula in Figure 2: (1) the scores for exposures, public health effects, and environmental effects are summed, (2) the scores for sensitive population and socioeconomic factors are summed; and (3) the two scores are multiplied to produce a cumulative impact score, which could range from 6 to 120. 

## 3. Results

**Figure 3:**
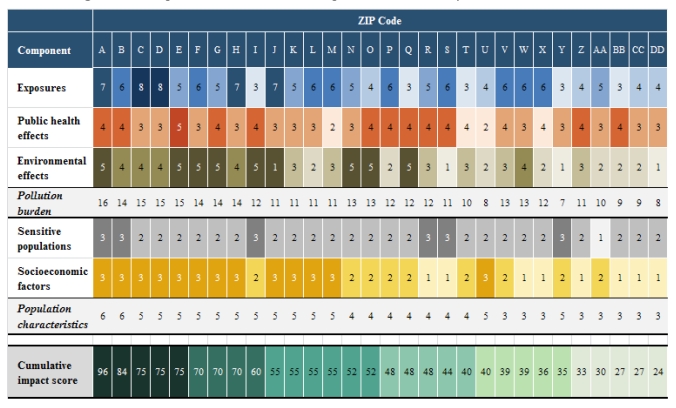
Component and cumulative impact scores for thirty California ZIP codes.

The results of the pilot analysis, including each component score and the total cumulative impact score, for the 30 ZIP codes are presented in Figure 3. The shading within each row (for each component and the cumulative impact score) indicates the magnitude of the score; the higher the score, the darker the shading. The table orders the ZIP codes from the highest (96) to lowest (27) cumulative impact score.

Here, ZIP code ‘A’ has the highest score, based on both a relatively high pollution burden and the highest possible score for population characteristics. None of the ZIP codes selected in the pilot had an exposure component score greater than 8. No ZIP codes were assigned the maximum scores for either exposure or environmental effects, indicating that none of them fell within the highest score of all ZIP codes statewide for either component. This may reflect a normalizing effect from using multiple indicators to represent a component. That is, it is relatively rare for all of the indicators to maximize in a given ZIP code. We note that this proposed screening method is aimed at identifying places impacted by multiple environmental and social stressors. For this reason, some communities facing substantial impact from a single stressor (such as an air pollutant), may not rank highly in overall cumulative impact score.

## 4. Discussion

The screening method was designed to be a science-based tool that is simple, transparent and understandable, and incorporates the key components of cumulative impacts, as specified in Cal/EPA’s working definition. The method yields scores that distinguish highly impacted from less impacted communities and identifies which of the components (exposures, public health effects, socioeconomic factors, *etc*.) are the primary contributors to a community’s cumulative impact score by visual inspection. A more statistical approach (e.g., correlations or factor analysis) may be appropriate when evaluating a broader dataset such, as a state- or region-level analysis.

A screening method, while not comprehensive, can evaluate one community’s impacts relative to others in the study. That is, a screening assessment does not produce an absolute measure of impact or predict human health risks, but rather the screening method is a ranking tool. Even though a screening method does not calculate risks, such a method can inform risk assessments by identifying uncertainties or new areas of concern for evaluation. A screening method cannot detect the impact of small incremental changes within a community nor can the method determine the cause of health outcomes in a community. While the screening may highlight the presence of certain public health effects in the community, the method does not elucidate the source of the health effects or its attribution to a specific exposure. The screening method is not intended to supplant existing regulatory requirements, such as those specified in the California Environmental Quality Act, but to provide another piece of information to assist decision-makers in achieving sustainable communities and preventing disproportionate impacts. 

An important future step is to refine and strengthen the methodology for conducting cumulative impact analysis. This will involve consideration of additional data sets not currently included and how the weighting and scoring of indicators may influence the outcomes. The process and criteria for choosing indicators will be further refined and the indicators influencing individual communities can be examined further. For example, if a community has a relatively high combined score, the data can be further examined to determine the influence of the various components. The components scores could also be further examined to determine which indicators are most influential for that component. 

Significant scientific and policy challenges will be faced when integrating considerations of cumulative impacts into program activities. A next step is to develop guidelines for conducting a screening cumulative impact analysis which identifies available relevant data and how they can be incorporated. Such guidelines would identify which indicators and databases are most useful as measures of the components (exposures, public health effects, environmental effects, sensitivity and socioeconomic factors). The guidelines could describe how the screening method can be adapted to different types of geographic areas, such as census tracts, cities and counties, or regional analyses. In addition to gathering relevant data, an important goal will be to make the data more accessible to communities and the public. Public and inter-agency input will be an important element of this work moving forward.

This screening method is intended to be useful to Cal/EPA and others who are integrating cumulative impacts considerations into their policies and programs. Identification of communities with the highest cumulative impact scores would allow Cal/EPA programs to target them for additional environmental monitoring, increased pollution enforcement activities, or to prioritize them for available incentive programs that reduce emissions or provide clean-up funds. When intra-agency efforts are needed to address multi-media impacts, the application of such a screening tool could assist in identifying impacted areas. This information could be used to target enforcement programs to reduce violations of existing laws and regulations and deter future violations in highly impacted communities. Screening for highly impacted communities could be used to prioritize outreach efforts to communities most in need of financial assistance. This assistance could be used to increase public participation opportunities and other capacity-building efforts. 

## 5. Conclusions

A substantial, growing body of scientific evidence raises concern for the cumulative impact of environmental pollutants, particularly for low-income and minority populations. The case for considering cumulative impacts in environmental decision-making is compelling. The screening methodology presented here provides a science-based tool that begins to facilitate this process. The methodology is simple and understandable. It is designed to incorporate information about contributors to cumulative impact in a community, both in terms of pollution burden and population characteristics. By allowing a screening analysis that distinguishes the more highly impacted communities, the methodology will assist environmental programs in addressing environmental justice concerns. Overcoming the significant scientific and policy challenges in integrating considerations of cumulative impacts into Cal/EPA’s program activities will require input from all interested parties. The screening methodology will help provide a focal point for such discussions. In addition to its value in environmental policy, it is hoped that the methodology will spur the development of new approaches and further stimulate research into the cumulative impacts of pollution.
